# Value of respiratory variation of aortic peak velocity in predicting children receiving mechanical ventilation: a systematic review and meta-analysis

**DOI:** 10.1186/s13054-019-2647-7

**Published:** 2019-11-22

**Authors:** Xiaoying Wang, Lulu Jiang, Shuai Liu, Yali Ge, Ju Gao

**Affiliations:** 1 Department of Anesthesiology, Clinical Medical College of Yangzhou University (Northern Jiangsu People’s Hospital), Yangzhou, 225001 China; 2 Department of Anesthesiology, The Second Xiangya Hospital of Central South University, Changsha, 410011 Hunan China; 3 Dalian Medical University, Dalian, 116044 Liaoning China

**Keywords:** Aortic, Pediatric, Velocity, Fluid, Ventilation

## Abstract

**Background:**

Accurate volume assessment is crucial in children under fluid therapy. Over the last decade, respiratory variation of aortic peak velocity (△VPeak) has been applied in intensive care unit and surgeries to help clinicians guide fluid management. The aim of this systematic review and meta-analysis was to test diagnostic performance of △VPeak in predicting fluid responsiveness of ventilated children and to explore the potential factors that influence the accuracy of △VPeak.

**Methods:**

We searched PubMed, Embase, and Cochrane from inception to April 2019 that evaluated association between △VPeak and fluid responsiveness after fluid challenge in children receiving mechanical ventilation. Data synthesis was performed within the bivariate mixed-effects regression model modified for synthesis of diagnostic test data.

**Results:**

Eleven studies with a total of 302 pediatric patients were included in our meta-analysis. The pooled sensitivity and specificity of △VPeak was 0.89 (95%CI = 0.77 to 0.95) and 0.85 (95%CI = 0.77 to 0.91), respectively. The diagnostic odds ratio (DOR) of △VPeak was 48 (95%CI = 15 to 155). SROC yielded an area under the curve of 0.91 (95%CI = 0.88–0.93). The △VPeak cutoff value was nearly conically symmetrical distribution and varied from 7 to 20%. After excluding several extreme studies, most data were centered between 12 and 13%. The medium and mean cutoff values of △VPeak were 12.2% and 12.7%, respectively. In subgroup analysis, compared to total data analysis, △VPeak performed weaker in the younger children group (mean ages < 25 months), with lower area under the summary receiver operating characteristic curve (AUSROC) of 0.80 (0.76 to 0.83), but stronger in the older children group (mean ages > 25 months), with AUSROC of 0.96 (0.94 to 0.97).

**Conclusions:**

Overall, △VPeak has a good ability in predicting fluid responsiveness of children receiving mechanical ventilation, but this ability decreases in younger children (mean age < 25 months). The optimal threshold of △VPeak to predict fluid responsiveness in ventilated children is reliable between 12 and 13%.

**Trial registration:**

The study protocol was registered prospectively on PROSPERO no. CRD42019129361.

## Background

Fluid resuscitation is the cornerstone of fluid management wherever in ICU or in perioperative period. Excessive volume expansion increases the risk of pulmonary edema, impairs the cardiac ventricular function, and even causes renal dysfunction [1]. However, hypovolemia could also lead to oxygen deliver disorder in critical organs which poses a great threat to life [2, 3].

Different from adult patients, pediatric patients possess larger ratio of body surface area to weight and higher water quality. Physically, children’s body compositions such as water proportion or muscle proportion are changing as they grow older, especially rapidly changing in preterm and term infants during the first 12 months of life [4]. Moreover, the myocardial structure of the heart, particularly the volume of cellular mass devoted to contractility, is significantly less developed in neonates than in adults. These differences, as well as developmental changes in contractile proteins, produce a leftward displacement of the cardiac function curve and less compliant ventricles. This developmental immaturity of myocardial structures also accounts for the tendency toward bi-ventricular failure, sensitivity to volume loading, poor tolerance of increasing afterload, and heart rate-dependent cardiac output [5, 6]. Due to pediatric patients’ special physical body structure, those deleterious effects caused by inappropriate fluid therapy should be taken more seriously in children. Therefore, physicians need to make a prompt and precise clinical decision in preloading judgment for children.

Considering special physical structure, it is hard to accurately assess children volume state in many situations. Clinically, many monitoring indices are applied to help physicians to assess fluid responsiveness. Traditional static indices, such as blood pressure (BP), central venous pressure (CVP), and pulmonary artery wedge pressure (PAWP), although are important but of limited sensitivity and specificity, especially in intra-abdominal hypertension, one lung ventilation, and prone position [7–9]. The famous Frank–Starling Law mandates that, in a certain range capability, the heart could adjust ventricular contractility and cardiac ejection to dynamic changes in ventricular filling [10, 11]. According to this, usually, a patient, who has the increment over 15% or 10% from baseline measurements in physiological parameters after volume expansion, is defined as fluid responsiveness positively [12–14]. Usually, indices which are directive to reflect cardiac ejection, such as stroke volume (SV), cardiac output (CO), cardiac index (CI), were seen as the gold standard indices to judge fluid responsiveness [14]. However, the measurement of the gold standard indices needs invasive operation and expensive monitoring equipments although they are accurate. Over the last decade, hemodynamic parameters, such as stroke volume variation (SVV) or pulse pressure variation (PPV), were reported to have a strong capability to assess fluid responsiveness during ventilation [15–17]. These hemodynamic parameters are based on heart-lung interaction that, in the air-tight thoracic construction, mechanical ventilation introduces cyclic changes in preload, leading to matched cardiac ejection and pulse pressure variation [18, 19]. However, the expensive equipment and invasive procedure also restrict the widespread application of these parameters.

Recently, noninvasive dynamic indices that calculate variation of peak velocity of the artery during several respiratory cycles measured by ultrasound have become an alternative quick volume assessment method [20, 21]. However, vessels in pediatric patients are thinner and more variable than adults. These distinctions bring challenges to noninvasive ultrasonagraphic techniques in children. Among all the vessels, the aortic artery is the largest and the nearest vessel next to cardiac ejection and is feasible and available to access whenever in ICU or in operating room. A recent review has reported that only respiratory variation in aortic blood flow peak velocity (△VPeak) has the capability to predict fluid responsiveness in children rather than SVV or PPV [22]. In 2015, a meta-analysis of 6 studies concluded that △VPeak showed good diagnostic accuracy in ventilated children [23]. However, it failed to find the optimal △VPeak threshold and potential factors that influence the accuracy of △VPeak due to limited included studies.

Our research was conducted to estimate the diagnostic accuracy of △VPeak as a predictor of fluid responsiveness in ventilated children, explore the potential factors that influence the accuracy of △VPeak, and analyze the most accurate threshold of △VPeak to predict fluid responsiveness in ventilated children.

## Methods

### Study selection and inclusion criteria

All diagnostic tests that study △VPeak as a predictor of fluid responsiveness of ventilated children were included in our research. The gold standard method of assessing fluid responsiveness was defined as an increase in CO, CI, SV, and stoke volume index (SVI) after volume expansion. And the sensitivity, specificity, and cutoff value of △VPeak to predict fluid responsiveness in ventilated children could be found or calculated in the original work.

Studies which did not clarify the patients’ age, number and basic characteristics, original paper or effective data unavailable, spontaneously breathing patients, studies conducted in animals or experimental researches, reviews, and letters were excluded. No language restriction was applied. No ethical and patient consent was required.

### Search strategy and data extraction

Two authors searched relevant clinical studies on PubMed, Embase, and Cochrane from inception to April 2019, and then independently screened literature, extracted data, and evaluated quality. If discrepancy existed, it was solved by the third arbitration. The whole search strategy used free words including fluid, responsiveness, pediatric, children, infant, kid, child, aortic, peak, and variation, and we combined words or phrases freely once more to acquire thorough results.

All the search was implemented by two authors strictly under the protocol, both of them screened the title and abstract. Irrelevant papers were neglected, while relevant and directly related papers were searched for full text, and those studies that measured △VPeak as the secondary observational index rather than the main index or the only index might also be included. Non-English language research was looked for original resources. Extracted data included three parts: the first one was basic information about the research such as the number of patients and study year. The second and the most important part was the results of the gold standard index and study index, including the gold standard index and the assessment of fluid responsiveness, cutoff value, sensitivity, specificity, and the area under receiver operating characteristic curve (ROC) of △VPeak. According to the collected data, we calculated true positive, false positive, false negative, and true negative values to construct the 2 × 2 contingency table. The last one was discrepancy among studies that could be the heterogeneity or potential factors that influence △VPeak. We collected age, liquid sort, measuring tool, ventilation parameter, vasoactive drugs, and study places.

All included researches were needed to provide adequate information satisfying meta-analysis. If data was unclear in the original paper to form 2 × 2 contingency table or characteristic form, we sent emails to the corresponding author for clarifying. If the author failed to respond in 1 month, we excluded the study in the final analysis.

### Quality assessment

Included studies were assessed by the Quality Assessment of Diagnostic Accuracy Studies-2 (QUADAS-2) recommended by the Cochrane Handbook [24, 25]. The QUADAS-2 tool consists of four domains: patient selection, index test, reference standard, and flow and timing. All domains were evaluated in terms of risk of bias and would be answered as “yes,” “no,” and “unclear” according to the specific content of each domain. The risk could be defined as low under the circumstance of a consistency of “yes,” and if all questions’ answers were “yes” but only one was “no,” the bias was possibly existing, and the author needs to judge the risk level according to the guidelines. “Unclear” was defined if the original study failed to provide adequate information that the authors had difficulty to judge. Quality assessment was performed by Revman software 5.3.

### Statistical analysis

Data synthesis was performed within the bivariate mixed-effects regression model that could incorporate the negative correlation, which might arise between the sensitivity and specificity as a result of the different threshold value of △VPeak used in studies [26, 27]. We estimated overall pooling of sensitivity, specificity, and diagnostic odds ratio (DOR) with 95% confidence interval (CI) using a bivariate random-effects model. We also plotted summary receiver operating characteristic curve (SROC) and calculated the area under the summary receiver operating characteristic curve (AUSROC).

Scatter plot was conducted to observe the distribution, dispersion, central tendency, and extremum of △VPeak cutoff value in all included studies. Mean and median △VPeak cutoff values were also calculated. We finally combined the mean, median, and scatter plot to estimate the optimal △VPeak threshold value for the prediction of fluid responsiveness in ventilated children.

Heterogeneity was quantitatively assessed by using the chi-square test and Cochran’s *Q* test. To describe the percentage of total attributed to heterogeneity rather than chance, we quantified the effect of heterogeneity using inconsistency (*I*^2^). *P* value for *Q* test < 0.1 or *I*^2^ > 50% were considered existing significant heterogeneity. Heterogeneity caused by the threshold effect in the diagnostic test was calculated in the Spearman correlation coefficient, which was estimated by the Moses–Shapiro–Littenberg [28]. Meta-regression was conducted to explore the potential sources of heterogeneity among studies beyond the threshold effect. Age, gold standard indices, fluid types, and vasoactive drugs were considered as potential factors to discriminate subgroups.

Public bias was estimated by Deek’s funnel plot asymmetry test, with *P* < 0.1 indicating statistical significance [29].

The results were expressed as mean (95%CI) or as mean ± standard deviation. Meta-analysis was performed by Stata 15. 0 (StataCorp, College Station, TX) with the Midas module. A two-tailed *P* < 0.05 was considered statistically significant.

## Results

### Characteristic of studies

**Fig. 1:**
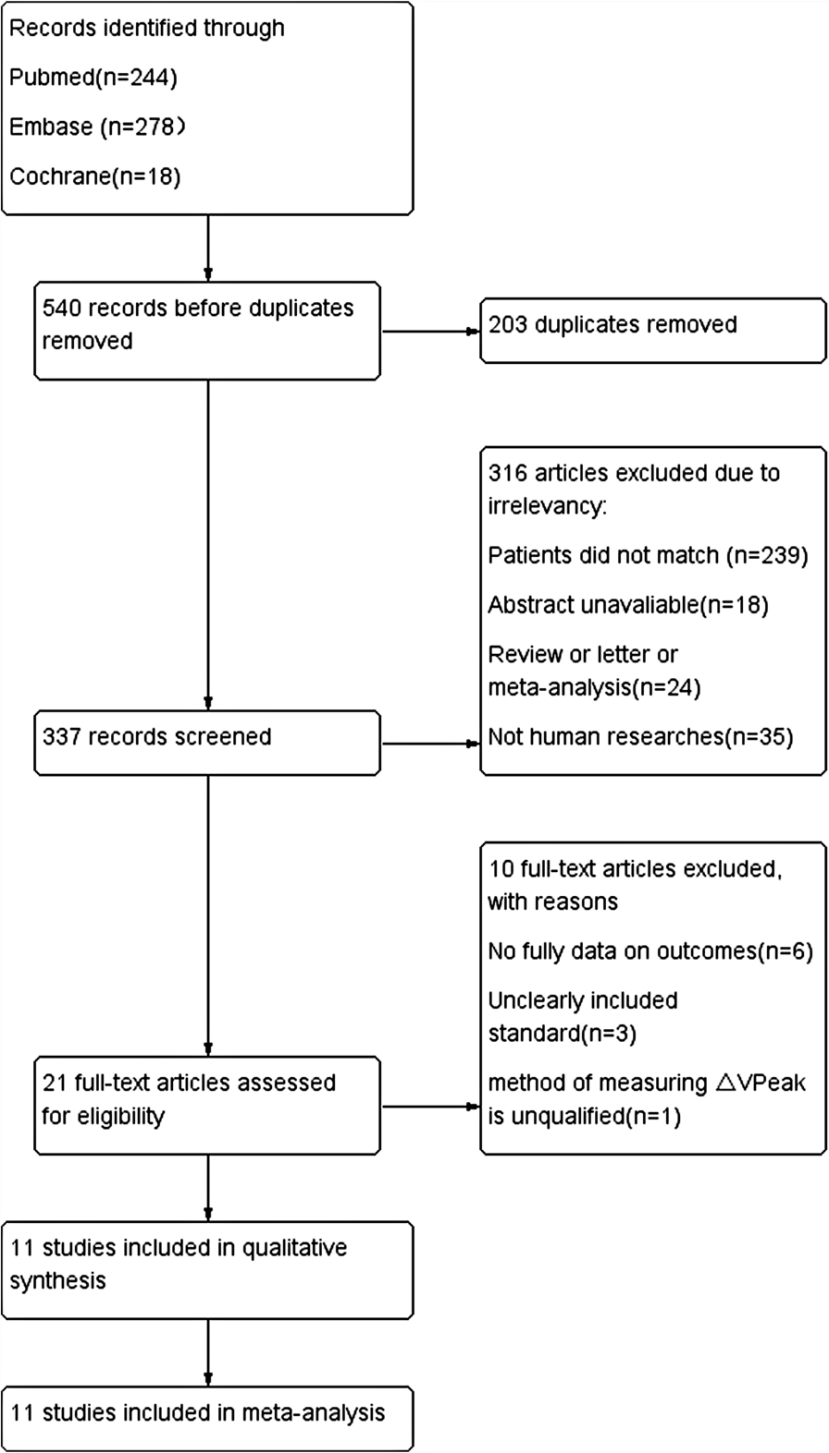
Flowchart of the study selection. △VPeak, respiratory variation of aortic peak velocity

Our meta-analysis yielded 540 studies after initially reviewed in three databases. Figure 1 shows the PRISMA selection of this meta-analysis. A total of 203 studies were removed due to duplicates. In the remaining 337 studies, 316 were excluded because of patients not eligible for the study purpose, abstract unavailable, reviews or letters, and animal researches. Further screening the full article, 10 full texts were excluded due to incomplete outcomes, unclearly included standard, and unqualified method of measuring △VPeak. Finally, 11 studies with a total of 302 pediatric patients were included in our meta-analysis [30–40].

Characteristics of the included studied are summarized in Tables 1 and 2. A total of 161 children patients were responders (53.3%) to fluid challenge. The cutoff value of △VPeak varied from 7 to 20%. Ten studies were performed before, during, or after cardiac surgery (*n* = 4) or neurosurgery (*n* = 3), and two of them took measurement time point after arriving at ICU for 30 min [35] and 1 h [31]. However, only one study [30] was performed in ICU with mixed diseases in critically ill children. Four studies referred △SV > 15%, and five studies referred △SVI > 15% as the gold standard index of responders to fluid challenge. Except Favia et al. [38], all other included studies were prospective diagnostic tests, and the gold standard index in this study was measured by peripheral artery wave contour analysis monitor rather than ultrasound. △VPeak in the total 11 studies was all performed with transthoracic echocardiography (*n* = 7) or transoesophageal echocardiography (*n* = 4).

**Table 1:** Selected characteristics of the included studies

No.	Authors/year	Sample size	Age (months)	Settings or time point of measure	VE	Golden index (responders)	Tool measuring golden index	Tool measuring △VPeak	Tide volume (ml/kg)	PEEP (cmH_2_O)	Vasoactive drugs
1	Durand 2008	26	*M* = 28.5	ICU	20 ml /kg/6%HES or saline 15–30 min	△SV > 15%	TTE	TTE	7.4	4	Yes
2	Choi 2010	21	*A* = 30	VSD repair, 1 h after arriving at ICU	10 ml/ kg/6%HES over 20 min	△SV > 15%	TTE	TTE	10	0	Yes
3	Renner 2011	27	*A* = 17	Before cardiac surgery begin	10 ml/ kg/6%HES	△SVI > 15%	TOE	TOE	10	3–5	Yes
4	Pereira 2011	30	*A* = 112	Before neurosurgery incision	20 ml /kg/saline over 15 min	△VTI > 15%	TTE	TTE	10	0–2	Yes
5	Byon 2013	33	*A* = 72.1	Stable during neurosurgery	10 ml/ kg/6%HES 10 min	△SVI > 10%	TTE	TTE	10	0	UC
6	Lee 2014	26	*A* = 28	VSD repair, 30 min after arriving at ICU	10 ml/ kg/6%HES 20 min	△SV > 15%	TTE	TTE	10	5	Yes
7	Lee 2015	29	*A* = 13.7	Once sternum closed after VSD repair	10 ml/ kg/6%HES 10 min	△SVI > 15%	TOE	TOE	10	UC	Yes
8	Krishna 2016	42	*A* = 76	General endotracheal anesthesia	10 ml/ kg/1%DRL 5-10 min	△SVI ≥ 15%	TTE	TTE	10	0	UC
9	Favia 2017	16	*A* = 24.2	Sternal closure in cardiac surgery	10 ml /kg/crystalloid or blood products	△CI > 10%	MostCare™	TOE	8–10	0–10	No
10	Lee 2017	30	*A* = 19.2	After cardiac surgery end before entering ICU	10 ml/ kg/6%HES 20 min	△SVI > 15%	TOE	TOE	10	UC	Yes
11	Morparia 2018	22	*A*==69.8	Neurosurgery	10 ml/ kg/crystalloid	△SV > 15%	TTE	TTE	8–10	0–4	sNO

*A* average age, *min* minute, *M* median age, *VE* volume experiment, *HES* hydroxyethe1 starch, *DRL* dextrose Ringer’s lactate, *△VPeak* respiratory variation of aortic peak velocity, *VSD* ventricular septal defect, *PEEP* positive end-expiratory pressure, *UC* unclear, *SV* stroke volume, *SVI* stroke volume index, *VTI* velocity-time integral, *TTE* transthoracic echocardiography, *TOE* transoesophageal echocardiography

**Table 2:** Diagnostic performance of pulse pressure variation from included studies

No.	Authors/year	Threshold^a^ (%)	TP	FP	FN	TN	Sens (%)	Spec (%)	AUROC	Responders (%)
1	Durand 2008	12	15	1	3	7	81.2	85.7	0.85	69.23
2	Choi 2010	20	10	1	1	9	91	90	0.83	52.38
3	Renner 2011	7	13	2	0	12	100	85	0.92	48.15
4	Pereira 2011	10	17	0	0	13	100	100	1.0	56.67
5	Byon 2013	11	13	5	2	13	86.7	72.2	0.804	45.45
6	Lee 2014	14	12	2	1	11	92.0	85.0	0.956	50.00
7	Lee 2015	13.5	9	3	4	13	69.2	78.6	0.77	44.83
8	Krishna 2016	12.2	24	1	0	17	100	94	0.975	57.14
9	Favia 2017	16.5	6	4	1	5	83.0	55.0	0.70	43.75
10	Lee 2017	12	10	2	7	11	58.8	84.6	0.765	56.67
11	Morparia 2018	12.3	10	1	3	8	77	89	0.90	59.09

*TP* true positive, *FP* false positive, *FN* false negative, *TN* true negative, *Sens* sensitivity, *Spec* specificity, *AUROC* area under the receive operator characteristic curve, *Threshold*^*a*^ threshold used in studies to achieve corresponding sensitivity and specialty, *95%CI* 95% confidence interval

### Methodological quality of studies

**Fig. 2:**
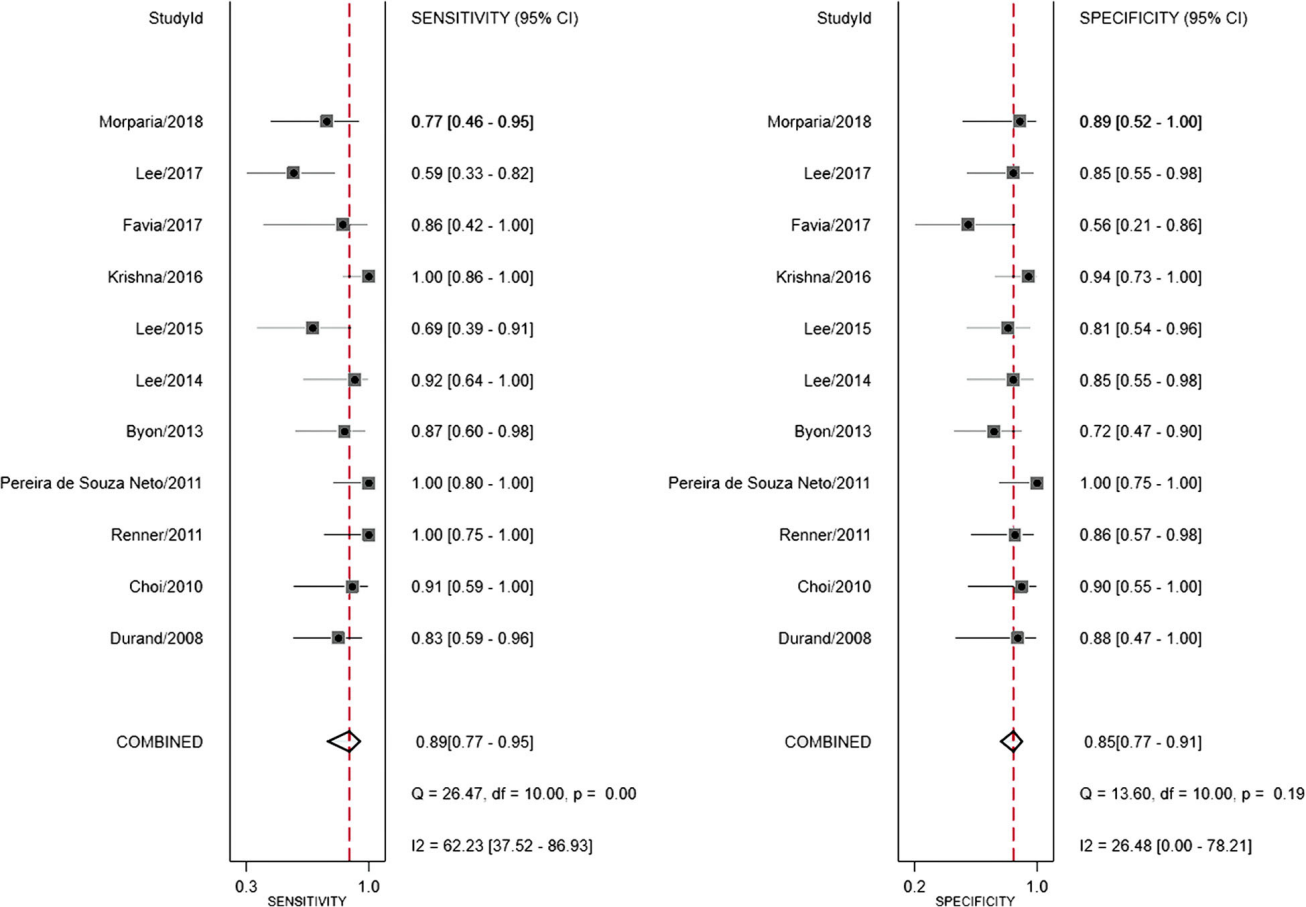
Sensitivity and specificity of respiratory variation of aortic peak velocity for prediction of fluid responsiveness in ventilated children for all data. CI, confidence interval

All included studies met patient spectrum criteria, had acceptable delays between index and reference tests, and performed the index and reference test in all included patients. One study used MostCare™ monitor to measure CI as a reference standard. Overall, the included studies were poorly reported in terms of blindness between index and reference tests, and most studies clarified uninterpretable tests unclearly. Table 3 and Fig. 2 show the methodological quality of the included studies, which was assessed by QUADAS2.

**Table 3:** Quality assessment of included studies using QUADAS-2 domains

Author/year	Risk of bias				Applicability concerns		
	Patient selection	Index test	Reference standard	Flow and timing	Patient selection	Index test	Reference standard
Durand 2008	☹	?	☺	☺	☺	☺	☺
Choi 2010	☺	?	☺	☺	☺	☺	☺
Renner 2011	☺	☺	☺	☺	☹	☺	☺
Pereira 2011	☺	☺	☺	☺	☺	☺	☺
Byon 2013	☺	?	☺	☺	☺	☺	☺
Lee 2014	☺	?	?	?	☺	☺	☺
Lee 2015	☺	?	?	?	☺	☺	☺
Krishna 2016	☺	?	☺	?	?	?	☺
Favia 2017	☺	?	☹	?	☺	☺	☺
Lee 2017	☺	?	☺	☺	☺	☺	☺
Morparia 2018	☺	?	☺	☺	☺	☺	☺

☺ low risk, ☹ high risk, ? unclear risk

### Prediction of fluid responsiveness based on △VPeak

**Fig. 3:**
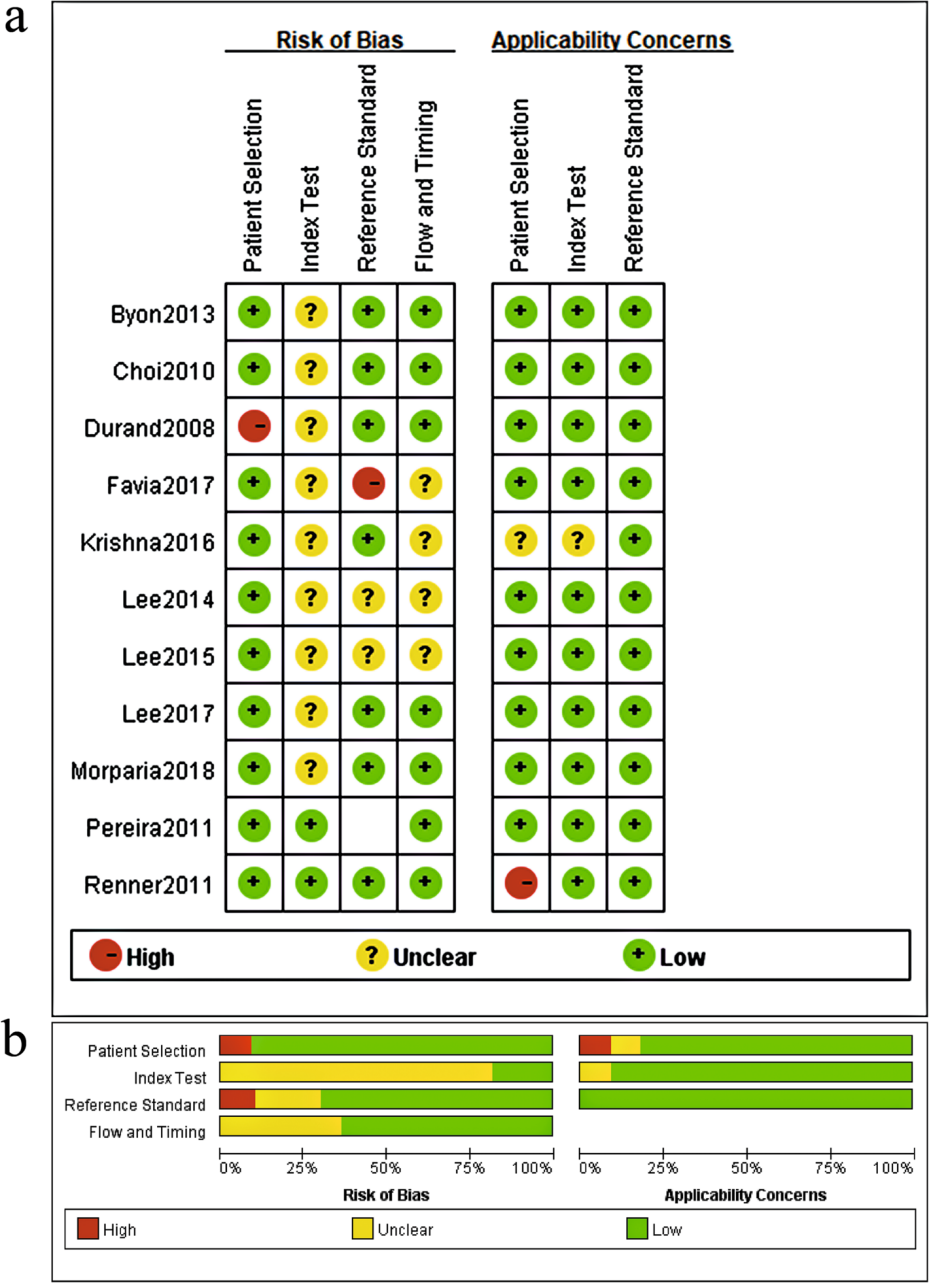
Risk of bias and applicability concerns for the studies included in the meta-analysis. **a** Risk-of-bias summary. **b** Risk-of-bias graph

**Fig. 4:**
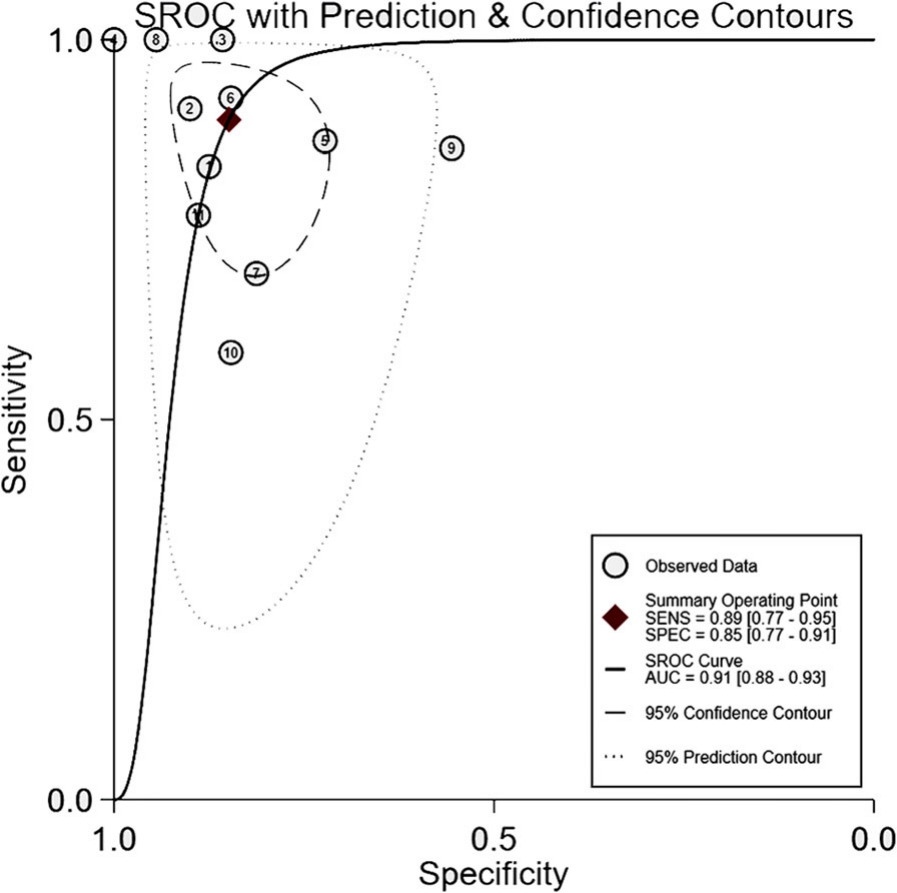
Summary receiver operating characteristic curve of respiratory variation of aortic peak velocity for predicting fluid responsiveness. Each circle represents individual study estimates. The diamond is the summary point representing the average sensitivity and specificity estimates. AUC, area under the curve; SENS, sensitivity; SPEC, specificity; SROC, summary receiver operating characteristics. The ellipses around this summary point are the 95% confidence region (dashed line) and the 95% prediction region (dotted line)

Pooled sensitivity of △VPeak was 0.89 (95%CI 0.77 to 0.95), and pooled specificity of △VPeak was 0.85 (95%CI 0.77 to 0.91), presented in Fig. 3. The positive and negative likelihood ratio of △VPeak was 5.9 (95%CI 3.6 to 9.7) and 0.12 (95%CI 0.05 to 0.29), respectively. The diagnostic odds ratio (DOR) of △VPeak was 48 (95%CI 15 to 155). SROC yielded an area under the curve of 0.91 (95%CI 0.88–0.93), presented in Fig. 4.

### The assessment optimal △VPeak threshold value to predict fluid responsiveness in ventilated children

**Fig. 5:**
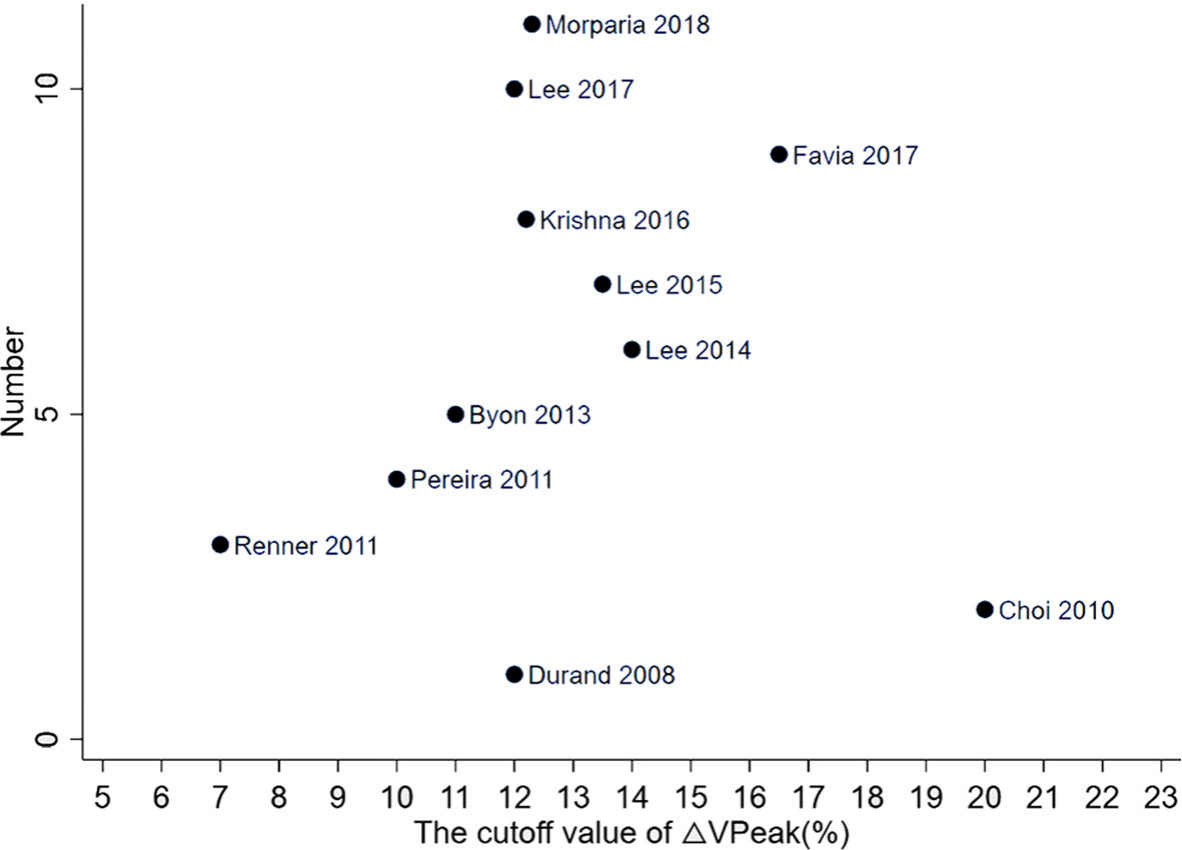
Scatter plot of cutoff value of respiratory variation of aortic peak velocity in included studies. The cutoff values of included studies are as follows: (1) Durand 2008 [30], 12%; (2) Choi 2010 [31], 20%; (3) Renner 2011 [32], 7%; (4) Pereira 2011 [33], 10%; (5) Byon 2013 [34], 11%; (6) Lee 2014 [35], 14%; (7) Lee 2015 [36], 13.5%; (8) Krishna 2016 [37], 12.2%; (9) Favia 2017 [38], 16.5%; (10) Lee 2017 [39], 12%; (11) Morparia 2018 [40], 12.3%

The △VPeak cutoff value in each included studies is presented in a scatter plot shown in Fig. 5. In Fig. 5, the △VPeak cutoff values were nearly conically symmetrical distribution, varied from 7 to 20%, and excluding several extreme studies, most data were centered between 12 and 13%. The mean △VPeak cutoff value was 12.7%, with the deviation of 0.03. The medium △VPeak cutoff value was 12.2%.

### Heterogeneity investigation

Heterogeneity of these studies was assessed with Cochran’s *Q* of 1.98 and overall *I*^2^ statistic of 0%. No significant heterogeneity was found for specificity, DOR, and positive and negative likelihood ratio. However, *I*^2^ statistic of sensitivity was 62.23% and Cochran’s *Q* of it was 26.27. The cutoff value of △VPeak varied from 7 to 20%, which could cause threshold effect heterogeneity. The Spearman correlation coefficient was 1, indicating that the proportion of heterogeneity likely due to the threshold effect was 100%. As a result, meta-regression was unnecessary.

### Subgroup analysis

**Fig. 6:**
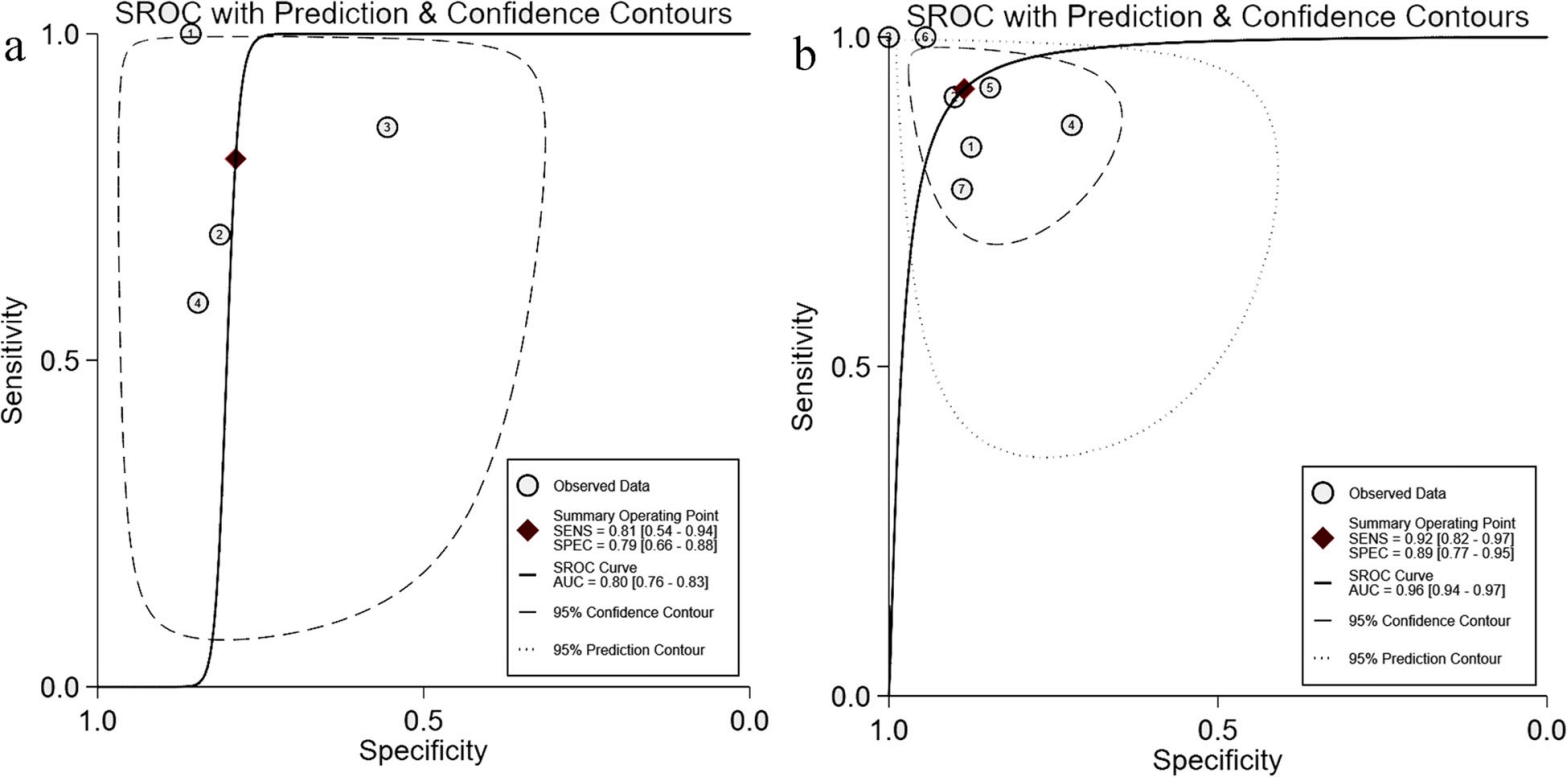
Summary receiver operating characteristics curve of respiratory variation of aortic peak velocity for the prediction of fluid responsiveness in **a** mean age of ventilated children < 25 months and **b** mean age of ventilated children ≥ 25 months. Circles represent each study included in the meta-analysis. AUC, area under the curve; SENS, sensitivity; SPEC, specificity; SROC, summary receiver operating characteristics

In subgroup analysis, we found that △VPeak performed weak in the younger children group, with the area under the curve of ROC of only 0.77, 0.70, and 0.765 in mean age of 13.7 months [36], 24.2 months [38], and 19.2 months [39], respectively. Compared to the total data, the younger children group (mean ages < 25 months) had lower AUSROC of 0.80 (0.76 to 0.83). However, △VPeak performed a stronger ability of prediction in the older children group (mean ages > 25 months), with AUSROC of 0.96 (0.94 to 0.97), presented in Table 4 and Fig. 6. Besides, the prediction accuracy of △VPeak was not affected by the gold standard index, vasoactive drugs, and fluid types (see Additional file 1).

**Table 4:** Diagnostic accuracy of △VPeak in the group of ages

Ages (mean)	Numbers	Sensitivity	Specificity	Diagnostic odds ratio	SROC (95%CI)
All patients	11	0.89 (0.77–0.95)	0.85 (0.77–0.91)	48 (15–155)	0.91 (0.88–0.93)
< 25 months	4	0.81 (0.54–0.94)	0.79 (0.66–0.88)	16 (4–64)	0.80 (0.76–0.83)
≥ 25 months	7	0.92 (0.82–0.97)	0.89 (0.77–0.95)	91 (21–385)	0.96 (0.94–0.97)

### Public bias

**Fig. 7:**
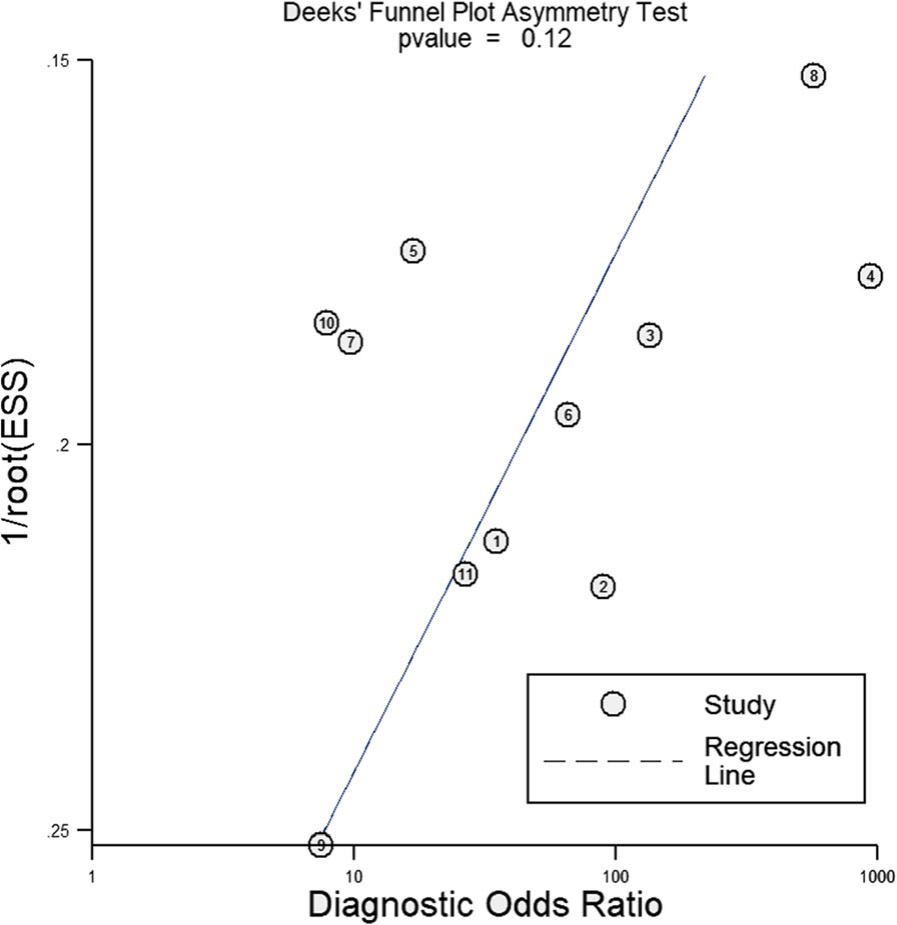
Deeks’ funnel plot with superimposed regression line. *P* value for slope coefficient is 0.12, which is greater than 0.05, suggesting the symmetry of the studies and the low likelihood of publication bias

Deek’s funnel plot asymmetry test of △VPeak is shown in Fig. 7, and no significant public bias was found in our meta-analysis (*P* = 0.12).

## Discussion

Our systematic review and meta-analysis found that overall, △VPeak performed well in predicting fluid responsiveness of ventilated children, with pooled sensitivity of 0.85, pooled specificity of 0.89, and AUSROC of 0.91. We synthesized the mean, median, and scatter plot to estimate that the most accurate value of △VPeak for prediction of fluid responsiveness in ventilated children is reliable between 12 and 13%. However, the accuracy of △VPeak decreased in children less than 25 months.

As we all know, each cardiac contraction brings a stroke volume from the left ventricle, and the blood flow passes through the vessel tract at different speed depending on the amount of blood ejection, the diameter of the tract, and blood velocity. In fact, △VPeak measured by ultrasound is similar to SVV, but it transforms invisible stroke volume to digital speed as an alternation. According to Rudnick’s theory, the closer the measurement position to cardiac ejection, the more accurate the prediction for hemodynamic indices to reflect central perfusion [41]. In our study, △VPeak showed a strong ability to predict fluid responsiveness in ventilated children, with AUSROC more than 0.9 and high sensitivity and specificity.

Desgranges et al. [23] first performed a six study systematic review and meta-analysis, but it failed to find optimal threshold value for △VPeak to predict fluid responsiveness in ventilated children due to extent variation of △VPeak cutoff value and inadequate data. However, more studies were published in the recent years, and comparing with demonstrating △VPeak is capable for predicting fluid responsiveness of ventilated children, acquiring optimal △VPeak threshold value of prediction possesses more clinical value. In a similar meta-analysis of respiratory variation in inferior vena cava diameter (△IVC), Si et al. [42] directly calculated the mean cutoff value of △IVC with only six studies in subgroups as the optimal threshold value of △IVC. In our scatter pot of △VPeak cutoff value, we observed that the cutoff value of △VPeak distributed symmetrically around the value of 12 to 13% [43]. And excluding several extreme studies, most data were centered between 12 and 13%. Combining mean and medium values, we estimated the most accurate value of △VPeak for prediction of fluid responsiveness in ventilated children is between 12 and 13%.

The extreme △VPeak cutoff values in our systematic review were 7% and 20%. Renner [32] performed volume expansion and △VPeak measurement after induction of anesthesia and before surgery. Without any fluid loss and hemodynamic alteration induced by surgery, such as trauma [44] and incision evaporation, patients possessed a stable volume state, and we observed a smaller value of △VPeak in Renner’s study than in other included studies, with 10.1% and 5.2% separately in responsive and non-responsive group before fluid loading, which could be the reason for only 7% cutoff value of △VPeak. Besides, fluid time is defined as the time of fast volume expansion with colloid 400–600 ml or crystalloid 300–1000 ml, usually within 30 min [45], while Renner’s study did not clarify fluid time of volume expansion. Recent evidence indicated that fluid responsive proportion decreased as fluid time extended [14], which could be a mixed factor for the cutoff value of △VPeak in Renner’s study. In another extreme condition, Choi et al. [31] got a 20% cutoff value for △VPeak. Compared to other included studies, Choi chose 1 h after arriving at ICU as measurement time point in cardiac surgery children. However, not only blood loss caused absolute inadequate volume state, trauma and stress could also rise plant nerve disorder, leading to relative hypovolemia [46, 47]. What is more, 1-h treatment in ICU may also increase variations in the hemodynamic state. Evidence shows that postoperative hemorrhage is a frequent complication after cardiac surgery [48], and patients after cardiac surgery represent a challenging high-risk population with specific requirements in catecholamine and fluid therapy in face of compromised cardiac performance and pronounced systemic inflammatory response after cardiopulmonary bypass [49]. Considering reasons above, patients were in high degree hypovolemia state before volume expansion, and we indeed observed a larger value of △VPeak in both groups, which could finally rise for large cutoff value of △VPeak.

In a subgroup analysis, compared to total data analysis, the prediction accuracy of △VPeak decreased in the younger age group (mean age < 25 months) and increased in the older age group (mean age > 25 months), indicating that clinicians should be cautious to apply △VPeak in younger children, especially children under 25 months, which was not mentioned in the previous study. Compared to Desgranges et al. [23], studies that gave a lower area under the curve of ROC (less than 0.8) emerged only in new studies published in recent years, and we have reasons to believe age is a potential factor to predictive accuracy of △VPeak. We consider inaccurate prediction of △VPeak could be relative to anatomical variation in younger children due to distinctive development, especially in children with congenital heart defects [50]. Studies reported that cardiac function in the immature left ventricle might be characterized by a higher basal contractile state, reduced compliance, and greater sensitivity to changes in afterload [51–53]. Moreover, Wolf et al. [53] found the neonatal myocardium is less responsive to preload and is vulnerable to overfilling. Due to limited data in different months phase, we failed to conclude the exact age group that optimally suitable for △VPeak. But children more than 3 years old could be more reliable on clinical work because of mature organs of the heart and vessels. With respect to the measurement tool of △VPeak, we are interested to find all younger group studies included in our research measured △VPeak by transthoracic or transoesophageal echocardiography. So far, no direct evidences have shown the distinction between transthoracic and transoesophageal echocardiography or the two methods could substitute for each other, but the closer measurement position to cardiac ejection, the more accurate to reflect central perfusion [41], and the two measurement methods both take close position to cardiac ejection. We concluded transoesophageal echocardiography had similar or slightly higher accuracy. Vasoactive drugs could activate α and β receptors in the heart and vessels, augment heart rate, and increase cardiac afterload, which could influence volume state and velocity of cardiac ejection. However, Sakai et al. [54] examined the effects of adrenaline administration on changes in circulatory dynamics and cardiac function in rats pretreated with chlorpromazine, and consequently found SV did not change significantly, and SVV significantly reduced at 1 and 2 min after treatment with adrenaline, but returned back to baseline thereafter. In our study, seven included studies used vasoactive drugs, and the vasoactive group yielded an area under SROC of 0.92, which was similar to total data, indicating vasoactive drugs had no significant impact on △VPeak.

In our study, overall data Cochran’s *Q* and *I*^2^ statistical analysis shown no significant heterogeneity, but significant heterogeneity for sensitivity existed and we still cannot rule out any potential heterogeneity. The threshold effect is one of the main causes of heterogeneity in diagnostic tests due to different cutoff values used in different studies to determine a positive (or negative) result. The Spearman correlation coefficient value in our study was 1, which meant the heterogeneity could be fully explained by the threshold effect [55, 56], which has not been explained in Desgranges’ study [23].

There are some limitations in our study. Firstly, sample size and study numbers in our meta-analysis were limited, although they were twice the number than previous studies. We included 11 diagnostic tests distributed between 2008 and 2018, with only 302 sample sizes, which were much smaller than the similar scale meta-analysis [42]. We attributed this phenomenon to the fact that ventilated children requiring △VPeak measurement were most critical or severe trauma, such as cardiac surgery, neurosurgery, or complicated diseases hospitalized in ICU. This population was rather small and each clinical study included in our meta-analysis collected limited samples. However, even under this condition, a meta-analysis still provided valuable information on the diagnostic accuracy until proven by larger or better-conducted studies [57]. Secondly, time of volume expansion was different or not clarified in our included studies. In the recent years, Toscani and colleagues [14] conducted a large sample meta-analysis finding that responsive proportion of population had no significant correlation with the type and volume of fluid but decreased with long infusion time, especially more than 30 min. Although most included studies in our meta-analysis restricted infusion time less than 20 min, we still could not exclude infusion time as a factor for △VPeak accuracy. Thirdly, except Favia et al. [38] using the peripheral artery wave contour analysis technique, the gold standard index was measured by transthoracic or transoesophageal echocardiography. To our knowledge, the closer measurement position to cardiac ejection, the more accurate prediction for hemodynamic indices to reflect central perfusion [41]. However, we failed to find difference after excluding Favia’s study (see Additional file 2), but we still could not rule out reference index measurement as a potential factor that influence accuracy of △VPeak, and more studies in the future could bring different results. Fourthly, included studies picked different time to start fluid challenge and △VPeak measurement, such as after anesthesia, during surgery, sternum closure, or even arriving at ICU. However, complicated operating procedure could rise variable hemodynamic state in different measurement points, which could not be neglected to our meta-analysis. Lastly, in subgroup analysis, we found age was a factor that influenced △VPeak accuracy. However, due to included studies providing a mean value of age rather than a limited scale of age, we only found a decline tendency of prediction accuracy of △VPeak rather than giving an accurate value of age that △VPeak should be recommended applied in. Therefore, more new studies are needed to be done in the future.

## Conclusions

Overall, △VPeak has a good ability in predicting fluid responsiveness of children receiving mechanical ventilation and the optimal threshold value of △VPeak to predict fluid responsiveness is reliable between 12 and 13%. However, we observed that this ability decreased in younger children, especially mean age less than 25 months; thus, we expect more studies to make a statistic treatment in the future.

## Supplementary information

**Additional file 1. **Subgroup analysis of the gold standard index, vasoactive drugs and fluid types. **Table S1.** Subgroup analysis of the gold standard index, vasoactive drugs and type of drugs. **Figure S1.** Summary receiver operating characteristics curve of respiratory variation of aortic peak velocity for the prediction of fluid responsiveness in subgroups of gold standard index, fluid types and vasoactive drugs.

**Additional file 2. **Excluded one retrospective study ‘s meta-analysis. **Figure S2**. Summary receiver operating characteristics curve of respiratory variations of aortic peak velocity for predicting fluid responsiveness in the left 10 studies (excluding Favia’s study [38]).

## Data Availability

The datasets used and/or analyzed in the present study are available from the corresponding author on reasonable request.

## Abbreviations

△VPeak: Respiratory variation of aortic peak velocity
AUSROC: Area under the summary receiver operating characteristic curve
BP: Blood pressure
CI: Cardiac index
CO: Cardiac output
CVP: Central venous pressure
DOR: Diagnostic odds ratio
PAWP: Pulmonary artery wedge pressure
PPV: Pulse pressure variation
QUADAS-2: Quality Assessment of Diagnostic Accuracy Studies-2
SROC: Summary receiver operating characteristic curve
SV: Stroke volume
SVI: Stoke volume index
SVV: Stroke volume variation

## References

[1] Marik PE, Monnet X, Teboul JL (2011). Hemodynamic parameters to guide fluid therapy. Ann Intensive Care.

[2] Bindels AJ, van der Hoeven JG, Graafland AD, de Koning J, Meinders AE (2000). Relationships between volume and pressure measurements and stroke volume in critically ill patients. Crit Care.

[3] Cecconi M, De Backer D, Antonelli M, Beale R, Bakker J, Hofer C (2014). Consensus on circulatory shock and hemodynamic monitoring. Task force of the European Society of Intensive Care Medicine. Intensive Care Med.

[4] Friis-Hansen B (1971). Body composition during growth. In vivo measurements and biochemical data correlated to differential anatomical growth. Pediatrics.

[5] Romero T, Covell J, Friedman WF (1972). A comparison of pressure-volume relations of the fetal, newborn, and adult heart. Am J Phys.

[6] Kirkpatrick SE, Pitlick PT, Naliboff J, Friedman WF (1976). Frank-Starling relationship as an important determinant of fetal cardiac output. Am J Phys.

[7] Malbrain MLNG, Waele JJD, Keulenaer BLD (2015). What every ICU clinician needs to know about the cardiovascular effects caused by abdominal hypertension. Anaesthesiol Intensive Ther.

[8] Kang WS, Oh CS, Park C, Shin BM, Yoon TG, Rhee KY (2016). Diagnosis accuracy of mean arterial pressure variation during a lung recruitment maneuver to predict fluid responsiveness in thoracic surgery with one-lung ventilation. Biomed Res Int.

[9] Drozdzynska MJ, Chang YM, Stanzani G, Pelligand L (2018). Evaluation of the dynamic predictors of fluid responsiveness in dogs receiving goal-directed fluid therapy. Vet Anaesth Analg.

[10] Sequeira V, Velden JVD (2015). Historical perspective on heart function: the Frank-Starling law. Biophys Rev.

[11] Cherpanath TG, Geerts BF, Lagrand WK, Schultz MJ, Groeneveld AB (2013). Basic concepts of fluid responsiveness. Neth Heart J.

[12] Cecconi M, Dawson D, Grounds RM, Rhodes A (2009). Lithium dilution cardiac output measurement in the critically ill patient: determination of precision of the technique. Intensive Care Med.

[13] Squara P, Cecconi M, Rhodes A, Singer M, Chiche JD (2009). Tracking changes in cardiac output: methodological considerations for the validation of monitoring devices. Intensive Care Med.

[14] Toscani L, Aya HD, Antonakaki D, Bastoni D, Watson X, Arulkumaran N (2017). What is the impact of the fluid challenge technique on diagnosis of fluid responsiveness? A systematic review and meta-analysis. Crit Care.

[15] Yang X, Du B (2014). Does pulse pressure variation predict fluid responsiveness in critically ill patients? A systematic review and meta-analysis. Crit Care.

[16] Ratti F, Cipriani F, Reineke R, Catena M, Paganelli M, Comotti L (2016). Intraoperative monitoring of stroke volume variation versus central venous pressure in laparoscopic liver surgery: a randomized prospective comparative trial. HPB (Oxford).

[17] Zhang Z, Lu B, Sheng X, Jin N (2011). Accuracy of stroke volume variation in predicting fluid responsiveness: a systematic review and meta-analysis. J Anesth.

[18] Michard F (2005). Changes in arterial pressure during mechanical ventilation. Anesthesiology..

[19] Michard F, Boussat S, Chemla D, Anguel N, Mercat A, Lecarpentier Y (2000). Relation between respiratory changes in arterial pulse pressure and fluid responsiveness in septic patients with acute circulatory failure. Am J Respir Crit Care Med.

[20] Yao B, Liu JY, Sun YB (2018). Respiratory variation in peripheral arterial blood flow peak velocity to predict fluid responsiveness in mechanically ventilated patients: a systematic review and meta-analysis. BMC Anesthesiol.

[21] Kim DH, Shin S, Kim N, Choi T (2018). Carotid ultrasound measurements for assessing fluid responsiveness in spontaneously breathing patients: corrected flow time and respirophasic variation in blood flow peak velocity. Br J Anaesth.

[22] Gan H, Cannesson M, Chandler JR, Ansermino JM (2013). Predicting fluid responsiveness in children: a systematic review. Anesth Analg.

[23] Desgranges FP, Desebbe O, Pereira de Souza Neto E, Raphael D, Chassard D (2016). Respiratory variation in aortic blood flow peak velocity to predict fluid responsiveness in mechanically ventilated children: a systematic review and meta-analysis. Paediatr Anaesth.

[24] Bossuyt PM, Leeflang MMG. Chapter 6: developing criteria for including studies. In: Deeks JJ, Bossuyt PM, Gatsonis C, editors. Cochrane handbook for systematic reviews of diagnostic test accuracy version 1. 0. 0: The Cochrane Collaboration; 2009.

[25] Leeflang Mariska M.G. (2008). Systematic Reviews of Diagnostic Test Accuracy. Annals of Internal Medicine.

[26] Field AP (2001). Meta-analysis of correlation coefficients: a Monte Carlo comparison of fixed- and random-effects methods. Psycho Methods.

[27] Riley RD, Abrams KR, Sutton AJ, Lambert PC, Thompson JR (2007). Bivariate random-effects meta-analysis and the estimation of between-study correlation. BMC Med Res Methodol.

[28] Moses LE, Shapiro D, Littenberg B (1993). Combining independent studies of a diagnostic test into a summary ROC curve: data-analytic approaches and some additional considerations. Stat Med.

[29] Deeks JJ, Macaskill P, Irwig L (2005). The performance of tests of publication bias and other sample size effects in systematic reviews of diagnostic test accuracy was assessed. J Clin Epidemiol.

[30] Durand P, Chevret L, Essouri S, Haas V, Devictor D (2008). Respiratory variations in aortic blood flow predict fluid responsiveness in ventilated children. Intensive Care Med.

[31] Choi DY, Kwak HJ, Park HY, Kim YB, Choi CH, Lee JY (2010). Respiratory variation in aortic blood flow velocity as a predictor of fluid responsiveness in children after repair of ventricular septal defect. Pediatr Cardiol.

[32] Renner J, Broch O, Gruenewald M, Scheewe J, Francksen H, Jung O (2011). Non-invasive prediction of fluid responsiveness in infants using pleth variability index. Anesthesia..

[33] Pereira de Souza Neto E, Grousson S, Duflo F, Ducreux C, Joly H, Convert J (2011). Predicting fluid responsiveness in mechanically ventilated children under general anaesthesia using dynamic parameters and transthoracic echocardiography. Br J Anaesth.

[34] Byon HJ, Lim CW, Lee JH, Park YH, Kim HS, Kim CS (2013). Prediction of fluid responsiveness in mechanically ventilated children undergoing neurosurgery. Br J Anaesth.

[35] Lee JY, Kim JY, Choi CH, Kim HS, Lee KC, Kwak HJ (2014). The ability of stroke volume variation measured by a noninvasive cardiac output monitor to predict fluid responsiveness in mechanically ventilated children. Pediatr Cardiol.

[36] Lee JH, No HJ, Song IK, Kim HS, Kim CS, Kim JT (2015). Prediction of fluid responsiveness using a non-invasive cardiac output monitor in children undergoing cardiac surgery. Br J Anaesth.

[37] Achar SK, Sagar MS, Shetty R, Kini G, Samanth J, Nayak (2016). Respiratory variation in aortic flow peak velocity and inferior vena cava distensibility as indices of fluid responsiveness in anaesthetised and mechanically ventilated children. Indian J Anaesth.

[38] Favia I, Romagnoli S, Di Chiara L, Ricci Z (2017). Predicting fluid responsiveness in children undergoing cardiac surgery after cardiopulmonary bypass. Pediatr Cardiol.

[39] Lee JH, Song IK, Kim EH, Kim HS, Kim JT (2017). Prediction of fluid responsiveness based on liver compression-induced blood pressure changes in children after cardiac surgery. Minerva Anestesiol.

[40] Morparia KG, Reddy SK, Olivieri LJ, Spaeder MC, Schuette JJ (2018). Respiratory variation in peak aortic velocity accurately predicts fluid responsiveness in children undergoing neurosurgery under general anesthesia. J Clin Monit Comput.

[41] Rudnick MR, Marchi LD, Plotkin JS (2015). Hemodynamic monitoring during liver transplantation: a state of the art review. World J Hepatol.

[42] Si X, Xu H, Liu Z, Wu J, Cao D, Chen J (2018). Does respiratory variation in inferior vena cava diameter predict fluid responsiveness in mechanically ventilated patients? A systematic review and meta-analysis. Anesth Analg.

[43] Irwin RJ, Irwin TC (2011). A principled approach to setting optimal diagnostic thresholds: where ROC and indifference curves meet. Eur J Intern Med.

[44] Roberts DJ, Leonard SD, Stein DM, Williams GW, Wade CE, Cotton BA (2019). Can trauma surgeons keep up? A prospective cohort study comparing outcomes between patients with traumatic brain injury cared for in a trauma versus neuroscience intensive care unit. Trauma Surg Acute Care Open.

[45] Rhodes A, Evans LE, Alhazzani W, Levy MM, Antonelli M, Ferrer R (2017). Surviving sepsis campaign: international guidelines for management of sepsis and septic shock: 2016. Crit Care Med.

[46] Milano R (2017). Fluid resuscitation of the adult trauma patient: where have we been and where are we going?. Nurs Clin North Am.

[47] Lynch T, Kilgar J, AL Shibli A (2018). Pediatric abdominal trauma. Curr Pediatr Rev.

[48] Lemaignen A, Birgand G, Ghodhbane W, Alkhoder S, Lolom I, Belorgey S (2015). Sternal wound infection after cardiac surgery: incidence and risk factors according to clinical presentation. Clin Microbiol Infect.

[49] Demirci C, Zeman F, Schmid C, Floerchinger B (2017). Early postoperative blood pressure and blood loss after cardiac surgery: a retrospective analysis. Intensive Crit Care Nurs.

[50] Williams P (2014). Acquired and congenital pediatric cardiac disease. Pediatr Ann.

[51] Rowland DG, Gutgesell HP (1995). Noninvasive assessment of myocardial contractility, preload, and afterload in healthy newborn infants. Am J Cardiol.

[52] Spotnitz WD, Spotnitz HM, Truccone NJ, Cottrell TS, Gersony W, Malm JR (1979). Relation of ultrastructure and function. Sarcomere dimensions, pressure-volume curves, and geometry of the intact left ventricle of the immature canine heart. Circ Res.

[53] Wolf AR, Humphry AT (2014). Limitations and vulnerabilities of the neonatal cardiovascular system: considerations for anesthetic management. Paediatr Anaesth.

[54] Sakai A, Sunada K (2017). Effects of adrenaline on circulatory dynamics and cardiac function in rats administered chlorpromazine. Odontology.

[55] Menéndez I, Derbyshire E, Carrillo T, Caballero E, Engelbrecht JP, Romero LE (2017). Saharan dust and the impact on adult and elderly allergic patients: the effect of threshold values in the northern sector of Gran Canaria, Spain. Int J Environ Health Res.

[56] De Winter JCF, Gosling SD, Potter J (2016). Comparing the Pearson and Spearman correlation coefficients across distributions and sample sizes: a tutorial using simulations and empirical data. Psychol Methods.

[57] Devillé WL, Buntinx F, Bouter LM, Montori VM, de Vet HC, van der Windt DA (2002). Conducting systematic reviews of diagnostic studies: didactic guidelines. BMC Med Res Methodol.

