# Supplementary figures and images for: Value of respiratory variation of aortic peak velocity in predicting children receiving mechanical ventilation: a systematic review and meta-analysis
Source: Crit Care. 2019 Nov 22;23:372. doi: 10.1186/s13054-019-2647-7 (PMC6874822; doi:10.1186/s13054-019-2647-7)

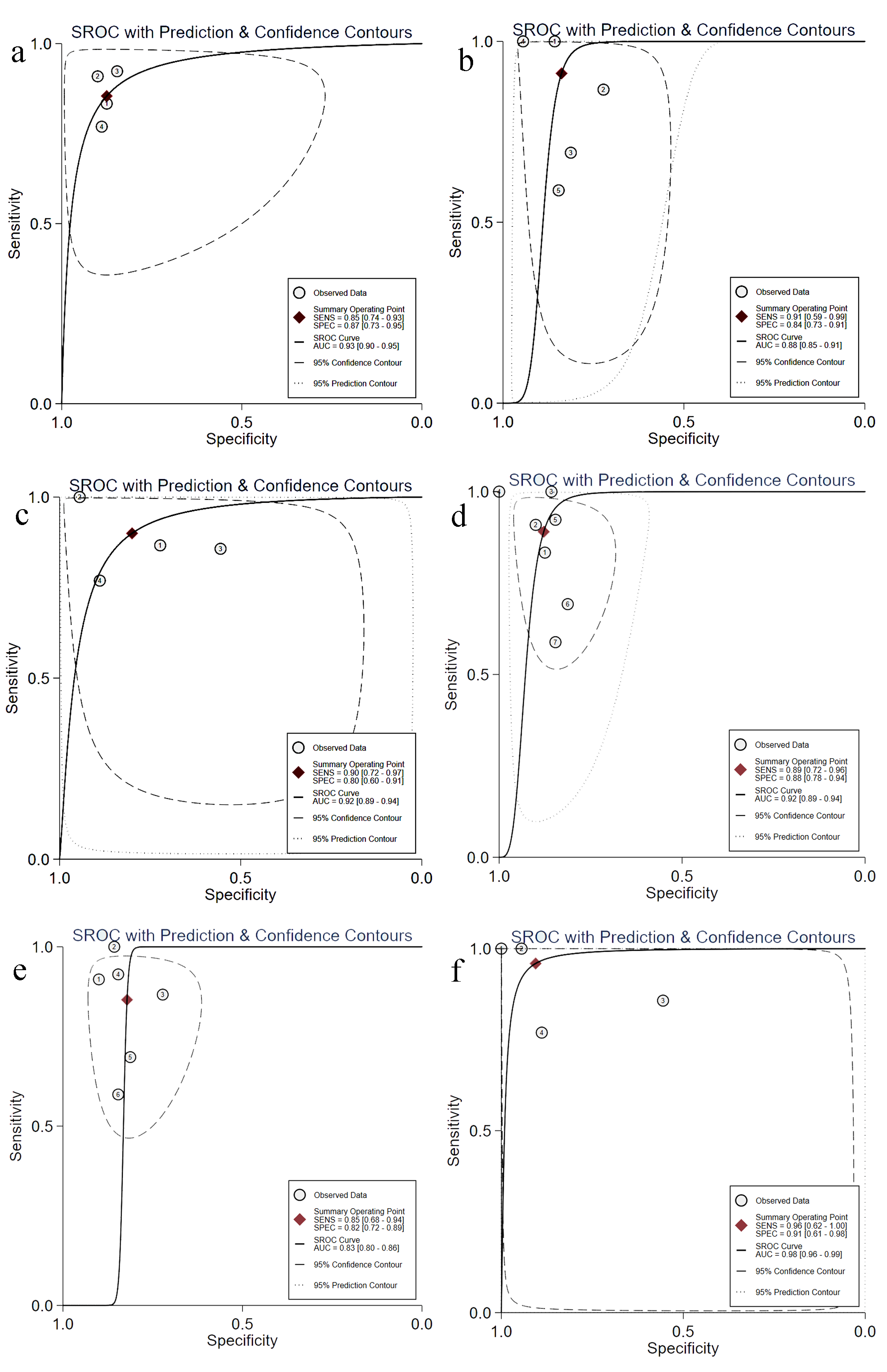

Supplement: Supplementary file 1 — Additional file 1. Subgroup analysis of the gold standard index, vasoactive drugs and fluid types. Table S1. Subgroup analysis of the gold standard index, vasoactive drugs and type of drugs. Figure S1. Summary receiver operating characteristics curve of respiratory variation of aortic peak velocity for the prediction of fluid responsiveness in subgroups of gold standard index, fluid types and vasoactive drugs. [file 13054_2019_2647_MOESM1_ESM.zip › Suppl.fig1.tif]

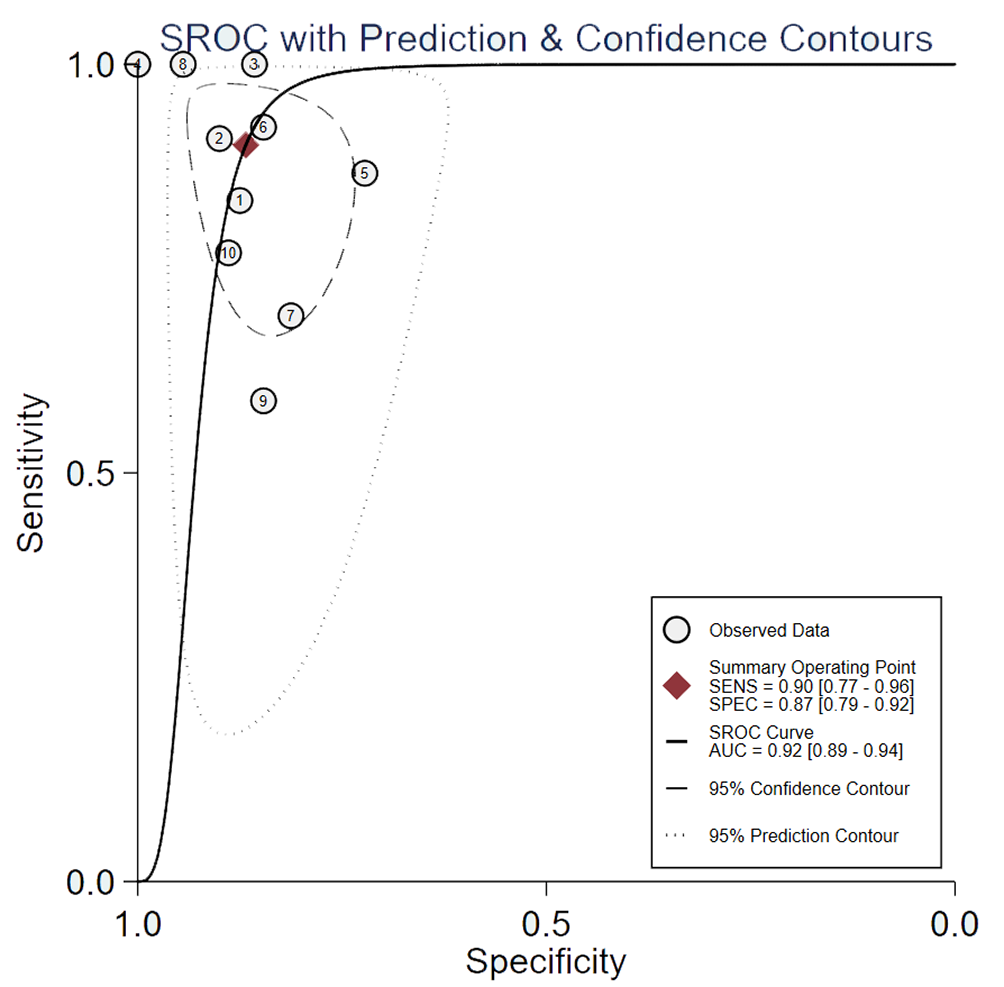

Supplement: Supplementary file 2 — Additional file 2. Excluded one retrospective study ‘s meta-analysis. Figure S2. Summary receiver operating characteristics curve of respiratory variations of aortic peak velocity for predicting fluid responsiveness in the left 10 studies (excluding Favia’s study [38]). [file 13054_2019_2647_MOESM2_ESM.tif]
