# Supplementary material for: Value of respiratory variation of aortic peak velocity in predicting children receiving mechanical ventilation: a systematic review and meta-analysis
Source: Crit Care. 2019 Nov 22;23:372. doi: 10.1186/s13054-019-2647-7 (PMC6874822; doi:10.1186/s13054-019-2647-7)
Supplement: Supplementary file 1 — Additional file 1. Subgroup analysis of the gold standard index, vasoactive drugs and fluid types. Table S1. Subgroup analysis of the gold standard index, vasoactive drugs and type of drugs. Figure S1. Summary receiver operating characteristics curve of respiratory variation of aortic peak velocity for the prediction of fluid responsiveness in subgroups of gold standard index, fluid types and vasoactive drugs. [file 13054_2019_2647_MOESM1_ESM.zip › Table Suppl 1.doc]

| Group | | N | sensitivity | specificity | Diagnostic Odds Ratio | SROC(95%CI) |
| --- | --- | --- | --- | --- | --- | --- |
| Golden index | SV | 4 | 0.85 (0.74-0.93) | 0.87 (0.73-0.95) | 41(12-137) | 0.93(0.90-0.95) |
|  | SVI | 5 | 0.91(0.59-0.99） | 0.84 (0.73-0.91) | 53 ( 5-534) | 0.88(0.85-0.91) |
| vasoactive drugs | Yes | 7 | 0.89(0.72- 0.96) | 0.88(0.78-0.94) | 58 (13- 263) | 0.92(0.89-0.94) |
|  | No or  Unclear | 4 | 0.90(0.72-0.97) | 0.80(0.60-0.91) | 36(6-234) | 0.92(0.89-0.94) |
| Type of fluid | crystalloid | 3 | - | - | - | - |
|  | colloid | 6 | 0.85(0.68-0.94) | 0.82( 0.72- 0.89) | 27 ( 8- 84) | 0.83 (0.80 - 0.86) |
| Total data | | 11 | 0.89(0.77-0.95) | 0.85(0.77-0.91) | 48(15-155) | 0.91(0.88-0.93) |

Table Suppl 1 :subgroup analysis of golden index,vasoactive drugs and type of drugs

-，stata software fails to calculate date less than 4 ;N,number
